# A Familial Cri-du-Chat/5p Deletion Syndrome Resulted from Rare Maternal Complex Chromosomal Rearrangements (CCRs) and/or Possible Chromosome 5p Chromothripsis

**DOI:** 10.1371/journal.pone.0076985

**Published:** 2013-10-15

**Authors:** Heng Gu, Jian-hui Jiang, Jian-ying Li, Ya-nan Zhang, Xing-sheng Dong, Yang-yu Huang, Xin-ming Son, Xinyan Lu, Zheng Chen

**Affiliations:** 1 Department of Medical Genetics, Zhongshan School of Medicine, Sun Yat-sen University, Guangzhou, PR China; 2 Guangzhou Women and Children’s Medical Center, Guangzhou, PR China; 3 Child Developmental Behaviour Center, The Third Affiliated Hospital, Sun Yat-sen University, Guangzhou, PR China; 4 Department of Infertility & Sexology, the Third Affiliated Hospital of Sun Yat-Sen University, Guangzhou, PR China; 5 Prenatal Diagnosis Center, Boai Hospital, Zhongshan, PR China; 6 Chaozhou Women and Children Hospital, Guangdong, PR China; 7 Department of Hematopathology, UT MD Anderson Cancer Center, Houston, Texas, United States of America; 8 Family Planning Research Institute of Guangdong, Guangzhou, PR China; 9 Department of Pediatrics, Baylor College of Medicine, Houston, Texas, United States of America; The Chinese University of Hong Kong, Hong Kong

## Abstract

Cri-du-Chat syndrome (MIM 123450) is a chromosomal syndrome characterized by the characteristic features, including cat-like cry and chromosome 5p deletions. We report a family with five individuals showing chromosomal rearrangements involving 5p, resulting from rare maternal complex chromosomal rearrangements (CCRs), diagnosed post- and pre-natally by comprehensive molecular and cytogenetic analyses. Two probands, including a 4½-year-old brother and his 2½-year- old sister, showed no diagnostic cat cry during infancy, but presented with developmental delay, dysmorphic and autistic features. Both patients had an interstitial deletion del(5)(p13.3p15.33) spanning ∼26.22 Mb. The phenotypically normal mother had *de novo* CCRs involving 11 breakpoints and three chromosomes: ins(11;5) (q23;p14.1p15.31),ins(21;5)(q21;p13.3p14.1),ins(21;5)(q21;p15.31p15.33),inv(7)(p22q32)dn. In addition to these two children, she had three first-trimester miscarriages, two terminations due to the identification of the 5p deletion and one delivery of a phenotypically normal daughter. The unaffected daughter had the maternal ins(11;5) identified prenatally and an identical maternal allele haplotype of 5p. Array CGH did not detect any copy number changes in the mother, and revealed three interstitial deletions within 5p15.33-p13.3, in the unaffected daughter, likely products of the maternal insertions ins(21;5). Chromothripsis has been recently reported as a mechanism drives germline CCRs in pediatric patients with congenital defects. We postulate that the unique CCRs in the phenotypically normal mother could resulted from chromosome 5p chromothripsis, that further resulted in the interstitial 5p deletions in the unaffected daughter. Further high resolution sequencing based analysis is needed to determine whether chromothripsis is also present as a germline structural variation in phenotypically normal individuals in this family.

## Introduction

Genomic imbalances such as chromosomal aberrations have been long recognized to be a major cause for genetic disorders, resulting in miscarriages, neonatal birth defects, postnatal developmental delay, autistic spectrum disorders and intellectual disability [1]. The phenotypes seen in each affected individual with chromosomal syndromes clearly depend on the particular chromosomal region with a variability in the clinical presentations [2]. Chromosome 5p deletion or Cri-du-chat syndrome (CDCs, MIM 123450) was first described by Lejeune in 1963 [3] and it is the one of most common chromosomal deletion syndrome in humans [4]. The incidence of CDCs is between 1∶50,000 to 1∶37000 live births [5]. The hallmark clinical features of CDCs include high-pitched cat-like monochromatic cry, microcephaly, a round face, hypertelorism, micrognathia, epicanthal folds, hypotonia, prominent nasal bridge, and severe psychomotor and intellectual disability [6]. Recurrent respiratory infections are also frequently observed in CDCs and pneumonia is the major cause of neonatal or infantile death [4], [7].

Approximately 80% of the CDCs patients carry a *de novo* 5p terminal or interstitial deletion and the majority of these deletions are paternal origin [6]. Less than 5% of the patients have *de novo* translocations or other rare chromosomal aberrations such as complex chromosomal rearrangements (CCRs) [8], [9]. About 10%–15% of the 5p deletions result from unbalanced segregation of a parental balanced rearrangement such as translocation or inversion [10], but very rarely from a balanced parental insertion [11] or CCRs [12].

CCRs are constitutional structural rearrangements involving more than three chromosomes with more than two breakpoints [13]. Typically, CCRs are three-way translocations with one breakpoint in each chromosome; however, CCRs with up to fifteen breakpoints have been reported [14]. Individuals with *de novo* CCRs, resulting in genomic imbalances at the chromosome breakpoints are frequently reported to have a high incidence of abnormal phenotypes and developmental delay/intellectual disability [15]. Heterozygous carriers of balanced CCRs are usually phenotypically normal, but have a significant risk [16] to have multiple spontaneous abortions [13] or chromosomally abnormal offspring [17].

Chromothripsis is a phenomenon in which tens to hundreds of genomic rearrangements occur in a one-off cellular crisis. Originally, it was observed in 2–3% of different cancer types [18]. Recent studies have shown that pediatric patients with constitutional abnormalities and *de novo* CCRs or complex genomic rearrangements (CGRs) also harbor chromothripsis [19], [20]. Using mate-paired sequencing along with molecular cytogenetic analyses in a family trio, including a proband with constitutional defects and CCRs of t(1;10;4)(p32.2;q21.1;q23) [19], a direct evidence of chromothripsis was found in two of the three chromosomes involved in the CCRs, and therefore, this catastrophic event most likely also has driven the *de novo* CCRs or CGRs in germline of the patients. However, such event has never been observed in phenotypically normal individuals.

Recent advances in clinical genetic settings have enabled integrated molecular and cytogenetic testing. The traditional chromosome analysis, Fluorescence *in situ* Hybridization (FISH), multiplex ligation-dependent probe amplification (MLPA), quantitative polymerase-chain-reaction (q-PCR) and short tandem repeats (STR) assay, and microarray based testing etc. are clinically utilized and have shown significant impact clinical human genetics, especially in diagnosing and counseling familial syndromes e.g. familial CCRs [21], [22] or familial 5p−/CDCs syndrome [23], [24].

Here, we report a 5p−/CDCs syndrome family with very rare and unique maternal CCRs, diagnosed by integrated molecular and cytogenetic analyses.

## Patients and Methods

### Patients and Family History

**Figure 1:**
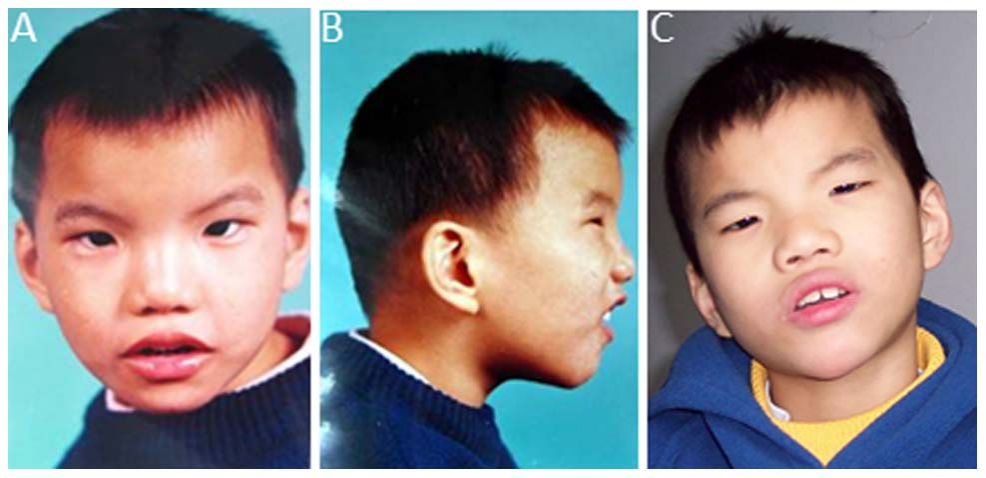
The male proband, A and B at age of 4 years and 6 months; C at age of 11 years and 9 months, showing microcephaly, micrognathia and hypertelorism, external canthus upslope, esostasis, plagiocephaly, short philtrum, large nasal bridge, and low-set ears.

This study was approved by medical ethics and institutional review board at Zhongshan School of Medicine, Sun Yat-sen University and, a consent signed by both parents was obtained. Probands were a four and a half-year-old male and a two and a half-year-old female born to healthy and unrelated parents. Both pregnancies were uneventful with no history of drug or alcohol usage during the pregnancies. The birth weight, length and head circumference on both probands were all within normal range in Chinese neonatal population. There were no cat-like cries observed at the birth in both probands. The male proband has incompletion cleft-palate, congenital hypertrophic pyloric stenosis, developmental delay and dysmorphic features, including round face, micrognathia and hypertelorism, external canthus upslope, plagiocephaly as well as some midfacial hypoplasia, short philtrum, large nasal bridge, and low-set ears (Figure 1). Simian creases were observed in both hands. In addition, he developed seizures at 8 months of age, and had neurodevelopmental abnormalities showing apraxia, callosum and alba dysplasia, cerebellum dysplasia, and ventriculomegaly. He raised his head at 9 months, crawled at 19 months and walked at 42 months and, had severe language delay with no speech till 38 months of age. Comparing with WHO 2006 reference, his circumference was 46.1 cm (−3SD) at 4 years and 6 months and 46.5 cm (−3SD) at age of 11 years and 9 months. The female proband had similar phenotypes including developmental delay, intellectual disability, as well as dysmorphic features. She died of pneumonia six months after the first genetic clinical visit.

**Figure 2:**
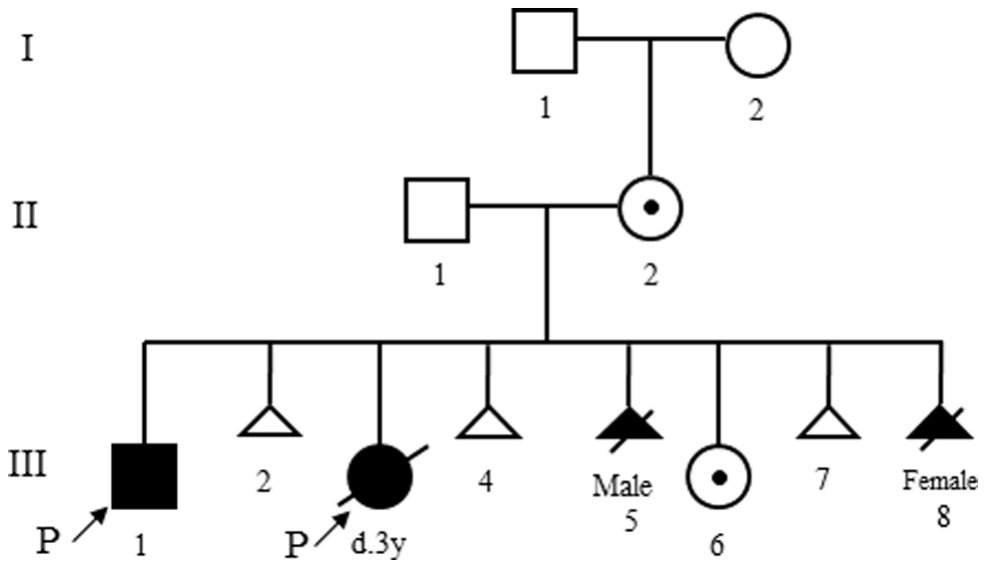
Pedigree. III 1, III 3, III 5 and III 8 all had identical 5p deletion. Prenatal diagnosis was performed on III 5, III 6 and III 8.□: normal male; ○: normal female; ⊙: carrier of ins(11;5); △: spontaneous abortion; ▴: affected and elected abortion; p↗: proband. “I” indicates the maternal grandparents of probands, and “II” indicates the parents of probands”.

The mother had a total of eight pregnancies (Figure 2).Notably, in between two probands, she also had a first trimester miscarriage. Genetic counseling was provided to the family after the first genetic visit of the probands. She subsequently had five additional pregnancies including two spontaneous abortions, two terminated pregnancies due to abnormal prenatal testing results and one delivery to a phenotypically normal daughter. This phenotypically normal daughter was followed up and evaluated for several years. At age of seven years, her IQ was 105 by WAIS intelligence test for children, which is average by comparing with the same age pediatric population in China.

### Conventional Cytogenetic Analysis

Chromosome analysis on peripheral blood (on I1, I 2, II1, II 2, III 1, III 3 and III 6), prenatal cord blood (on III5) and amniocytes (on III6, III 8) (Figure 2) were performed following the standard clinical cytogenetics laboratory protocols [25], [26]. The banding resolutions were ∼550 and ∼400 for blood samples and amniocytes, respectively. At least twenty metaphases were analyzed and chromosomal results were interpreted and reported according to the international system for human cytogenetic (ISCN 2005 and 2009) nomenclature.

### Short Tandem Repeats (STR) Analysis

Genomic DNA from peripheral blood and amniocytes were extracted using a QIAamp DNA Blood Maxi Kits (QIAGEN, Germany) and QIAamp DNA Mini Kits (QIAGEN, Germany), respectively.

STR markers within or in the vicinity of 5p13.3–p15.33 region were selected. (http://repeatmasker.genome.washington.edu/cgi-bin/RepeatMasker; http://www.genlink.wustl.edu/genethon_frame/chr5/chr5.html) and http://genome.ucsc.edu. Total 19 polymorphic STR markers, including four at 5p13.3, two at 5p14.1 and thirteen at 5p15.1–p15.33 were tested using specific 5′-labelled fluorescent primers (Table 1).

**Table 1:** Microsatellite markers with corresponding genome position and analysis results of 5p- case.

Marker	Band^a^	Position^a^	Primer Sequence^a^	Distance	Result
D5S1981	5p15.33	1154414–1155177	L-CCTGTACCAATCCATGCR-GAGCCATGTGAGTGTCC	224–268 bps	**+**
D5S417	5p15.33	3121203–3121461	L-TGGAAACTATGTATCTTGGAGGR-GCTGGCTTTAGGGTGG	90–104 bps	**+**
D5S406	5p15.32	4994043–4994367	L-CCTGCCAATACTTCAAGAAAR-GGGATGCTAACTGCTGACTA	160–186 bps	ND
D5S635	5p15.31	6312580–6312938	L-TAACATCCTCCAGGGCR-GTCCATTACATCACAGTTACTTT	159–170 bps	ND
**D5S676**	**5p15.31**	**7439488–7439901**	L-ATCTCTACCTGGCCCCR-CCATTATTCCATTTTGTTTG	228–239 bps	−
**D5S1953**	**5p15.31**	**7658252–7658591**	L-CAGCAGAGTGAGACTCCATR-TTCCTCAACTGAAGTTTCTGT	248–268 bps	−
**D5S580**	**5p15.31**	**8140533–8140841**	L-TAGTCTCTTCATGACTTGGTAR-CTGCATTCTAGCCTGGGC	147–187 bps	−
**D5S1957**	**5p15.31**	**8496820–8497172**	L-GGCTGATTGGTGAAGGACR-GGTTTGTAGATCTCCATTTCTG	205–213 bps	−
**D5S2095**	**5p15.31**	**9381790–9382151**	L-ATGAGCCACCATGCCTR-TCAAGGATAGTGATGCCATT	141–183 bps	−
**D5S630**	**5p15.31**	**9560963–9561368**	L-CATGACGATGTGGGCAGR-CCTTTCAGTGTAGAAGTGTGTGTGT	229–333 bps	−
**D5S2004**	**5p15.2**	**10500156–10500460**	L-TAGCCCAGGAGGTTGAGR-AACATGGAATCAAGATTTATTGAC	197–215 bps	−
**D5S416**	**5p15.1**	**16719995–16720360**	L-CTGGGGCTGTTTGTCAR-AGTGAAACTCGGNCCCTA	282–292 bps	−
**D5S2096**	**5p15.1**	**17447377–17447655**	L-TTGACTGTGACTTGAGAGGAR-GAAGCAGTATCCTTAGGGGA	196–210 bps	−
**D5S2113**	**5p14.1**	**26776388–26776731**	L-TTGATGAATCTCATTATGTTCACR-GCTAAATGTTTCCTTGGTCTT	221–265 bps	−
**D5S385**	**5p14.1**	**27460088–27460279**	L-CCTTGAGGCTCTCTTAAGGTR-AGAATAATAAAGCAGAACCCT	145–158 bps	−
**D5S2061**	**5p13.3**	**29976433–29976793**	L-TTTTGCCTGCAATTTAGTCAR-CCCATCGTGGAGTTTCAT	252–264 bps	−
D5S2854	5p13.3	30109262–30109579	L-CTTTTGGGAAACAGAAGCAAR-CTACAGATGGTACAGTGTAGGACG	157–175 bps	ND
D5S819	5p13.3	30873711–30874062	L-GTCACCCAAAAGTCATGAGGR-TGTACCCGCATGCTATACAA	259–286 bps	**+**
D5S1993	5p13.3	31705153–31705564	L-TCAGTGGAACTCAGGAGGR-AGACGGTAAACTTCTGGAGG	144–190 bps	**+**

a Position is based on the Feb. 2009 UCSC sequence; “+” for present, “−” for deletion, “ND” for not determined, deleted markers in bold.

Polymerase-chain-reactions (PCR) were carried out on II 1, II 2 and III1 and prenatally on III-6, III-8 using 5 ng of DNA, 2.5 mM MgCl2, 10 mMTris-HCl pH 8.3, 50 mMKCl, 250 mM each dNTP, 0.625 pmol of each primer, 0.25 u HotstarTaq DNA polymerase (QIANGEN GmbH, Hilden, Germany) in a total reaction volume of 5 ul. All PCRs were performed on a GeneAmp 9700 Therm cycle (PE Applied Biosystem), an initial denaturation 96°C for 5 min was followed by 35 cycle of 30 s at 95°C, 30 s at a prime-specific annealing temperature and 40 s at 72°C. The final extension was at 72°C for 10 min. PCR products were analyzed on an ABI PRISM 3100 Genetic Analyzer (PE Applied Biosystems). Allele sizes and peak areas were determined using GeneScan version 3.1, Genotype version 2.1 and LINKAGE version 5.1 (Applied Biosystems, CA).

The genotypes at each locus were examined in the tested subjects and compared with the parental genotypes to determine whether there was non-Mendelian segregation or an apparent deletion (i.e. absence of a maternal or paternal allele).

### Oligonucleotide Array Comparative Genomic Hybridization (array CGH) and Data Analysis

Oligonucleotide array CGH was performed on the male proband (III 1 at 12 years and 9 months old) and his phenotypically normal younger sister (III 6 at 6 years and 1 month old), as well as, both parents (II 1, II 2) using oligonucleotide-based arrays containing 180,000 probes from Agilent Technologies (Santa Clara, CA) according to manufacturer’s instruction, for a whole genome copy number analysis. Oligonucleotides probes on this 4x180K array were annotated against NCBI Build 37 (hg19). Array CGH image files were quantified using Agilent Feature Extraction software (version 10.5). Text file outputs containing quantitative data were imported into the Nexus copy number analysis software (Biodiscovery, Segundo, CA). Log2 ratio >0.2 was defined as gain and <−0.5 was defined as loss and all other ratios as normal.

### Fluorescence in Situ Hybridization (FISH)

FISH analyses were performed according to published protocols [27] in order to confirm chromosome abnormalities, STR and array CGH results. A total of twenty-one bacterial artificial chromosome (BAC) clones (RP11-59C22, RP11-810B19, RP11-473F9, RP11-1029M14, RP11-976O8, RP11-125A21, RP11-241M9, RP11-1122G9, RP11-420J19, RP11-23D12, RP11-349J3, RP11-373F8, RP11-1022E23, RP11-100I1, RP11-1055J13, RP11-428C17, RP11-62K5, RP11-106P5, RP11-318A6, RP11-1005N9, and RP11-876N17) mapping to 5p13.3–p15.32 region and three BAC clones (RP11-1029D4, RP11-916H5, and RP11-945C11) mapping to chromosome 21q21.1–q22.11 were selected from the University of California-Santa Cruz (UCSC), with detailed corresponding linear map position available in genome (Table 2). No BAC clones are available in the genomic region of 5p14.2. BAC clones were purchased from the Children’s Hospital Oakland Research Institute in Oakland, California, USA. BAC DNA extraction was prepared using Qiagen Large-Construct Kit (QIAGEN, Germany). DNAs are directly labeled using nick translation (Vylsis) according to the manufacturer’s instructions. Hybridized slides were analyzed using an Olympus BX60 fluorescence microscope equipped with appropriate filters and a LUCIA Cytogenetics 4.81 image analysis system.

**Table 2:** FISH results using BAC clones.

Clone-ID	Band	Genome position	FISH signals					
			Proband	Daughter		Mother		
			der(5)	der(5)	der(11)	der(5)	der(11)	der(21)
RP11-59C22	5p15.32	5126675–5299266	−	−	−	−	−	+
RP11-810B19	5p15.32	5340650–5533248	−	−	−	−	−	+
RP11-473F9	5p15.32	5557914–5764620	−	−	−	−	−	+
RP11-1029M14	5p15.32	5916166–6102533	−	−	−	−	−	+
RP11-976O8	5p15.31	6507087–6708095	−	−	−	−	−	+
**RP11-125A21**	**5p15.31**	**6972214**–**7133324**	−	−	+	−	+	+
**RP11-241M9**	**5p14.3**	**18782135**–**18942612**	−	−	+	−	+	−
**RP11-1122G9**	**5p14.3**	**22898936**–**23029586**	−	−	+	−	+	−
**RP11-420J19**	**5p14.3**	**23043359**–**23193686**	−	−	+	−	+	−
**RP11-23D12**	**5p14.3-14.2**	**23276737**–**23446734**	−	−	+	−	+	−
**RP11-349J3**	**5p14.1**	**26918160**–**27108056**	−	−	+	−	+	−
**RP11-373F8**	**5p14.1**	**27102060**–**27277931**	−	−	+	−	+	−
**RP11-1022E23**	**5p14.1**	**27163816**–**27326620**	−	−	+	−	+	−
**RP11-100I1**	**5p14.1**	**27240267–27406644**	−	−	+	−	+	+
RP11-1055J13	5p14.1	27368992**–**27556019	−	−	−	−	−	+
RP11-428C17	5p14.1	27881699**–**28063168	−	−	−	−	−	+
RP11-62K5	5p14.1	28461001**–**28639267	−	−	−	−	−	+
RP11-106P5	5p14.1	28558137–28724074	−	−	−	−	−	+
RP11-318A6	5p14.1	28946908–29130606	−	−	−	−	−	+
RP11-1005N9	5p13.3	30402929–30590905	+	+	−	+	−	−
RP11-876N17	5p13.3	31289933–31479969	+	+	−	+	−	−
RP11-1029D4	21q21.1	22201380–22403208	−	−	−	−	−	+
RP11-916H5	21q21.3	29708505–29916045	−	−	−	−	−	+
RP11-945C11	21q22.11	31551625–31745727	−	−	−	−	−	+

FISH results using the BAC clones with corresponding genome position on chromosome 5p and 21q, demonstration the deletions in the male proband and in the normal sister, as well as the insertion of ins(11;5) in the normal sister and the mother, and two ins(21;5) in the mother. “+” indicates FISH signal present and “−” indicates FISH signal not present on the correlated chromosomes. Abnormal chromosomes are in bold.

“+”, positive for FISH signal; “−”, negative for FISH signal.

## Results

### Chromosome Analysis

**Figure 3:**
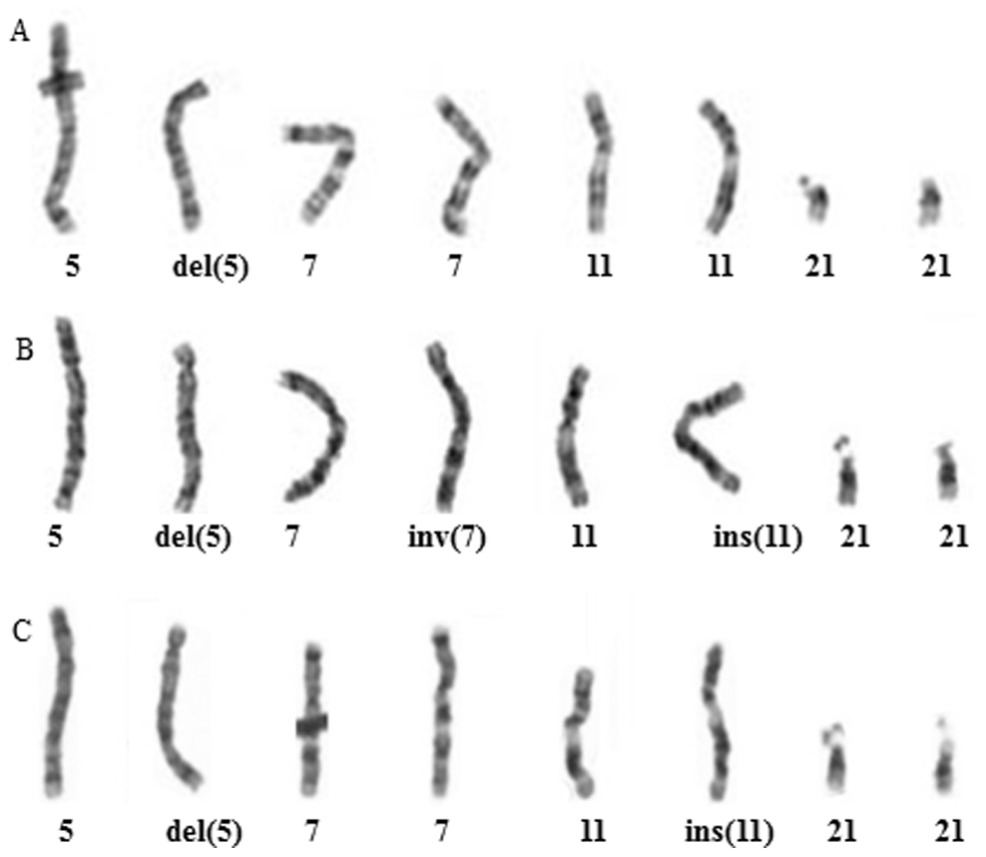
Partial karyotypes of the proband III 1(A) showing del(5)(p13.3p15.3); the mother II 2 (B) showing apparently ins(11;5)(q23;p14.1p15.3),inv(7)(p22q32); ins(21) was cryptic cytogenetically, and sister III 6 (C) showing ins(11;5)(q23;p14.1p15.3) only.

Chromosome analysis of peripheral blood on the two probands (III 1, III 3) revealed an identical interstitial deletion of chromosome 5p13.3p15.3. A clinical diagnosis of CDCs or chromosome 5p deletion syndrome was made based on chromosomal analyses and clinical manifestations in both probands. Subsequently, this family was provided with genetic counseling. Chromosome studies on both parents were performed and revealed the phenotypically normal mother to carry an apparently complex karyotype of 46,XX,ins(11;5) (q23;p13.3p15.3),inv(7)(p22q32) showing an apparently one balanced insertion between chromosomes 5 and 11, and a pericentromeric inversion 7, whereas the father had a normal karyotype. Further chromosome analysis on maternal grandparents confirmed the abnormal finding in the mother to be a *de novo* event. Due to the 5p deletions in both probands, the miscarriage of III 2 and the complex karyotype in the mother, this couple agreed to perform prenatal diagnosis for all future pregnancies. Prenatal chromosome analysis was performed on III 5 using cord blood at 21 week gestational age, and on III 6 and III 8 using amniotic fluid at 19 week gestational age (Figure 2). III 5 and III 8 showed abnormal karyotypes of 46,XY,del(5)(p13.3p15.3)mat and 46,XX,del(5)(p13.3p15.3),inv(7)(p22q32)mat, respectively, and both pregnancies were elected for termination by parents after genetic counseling. III 6 showed an apparently balanced insertion of 46,XX,ins(11;5)(q23;p13.3p15.3)mat. Additional prenatal molecular based testing was recommended to confirm the chromosome result. Partial karyotypes of the male proband, the mother and the phenotypically normal sister are shown in Figure 3 A, B and C, respectively.

### STR Results

**Figure 4:**
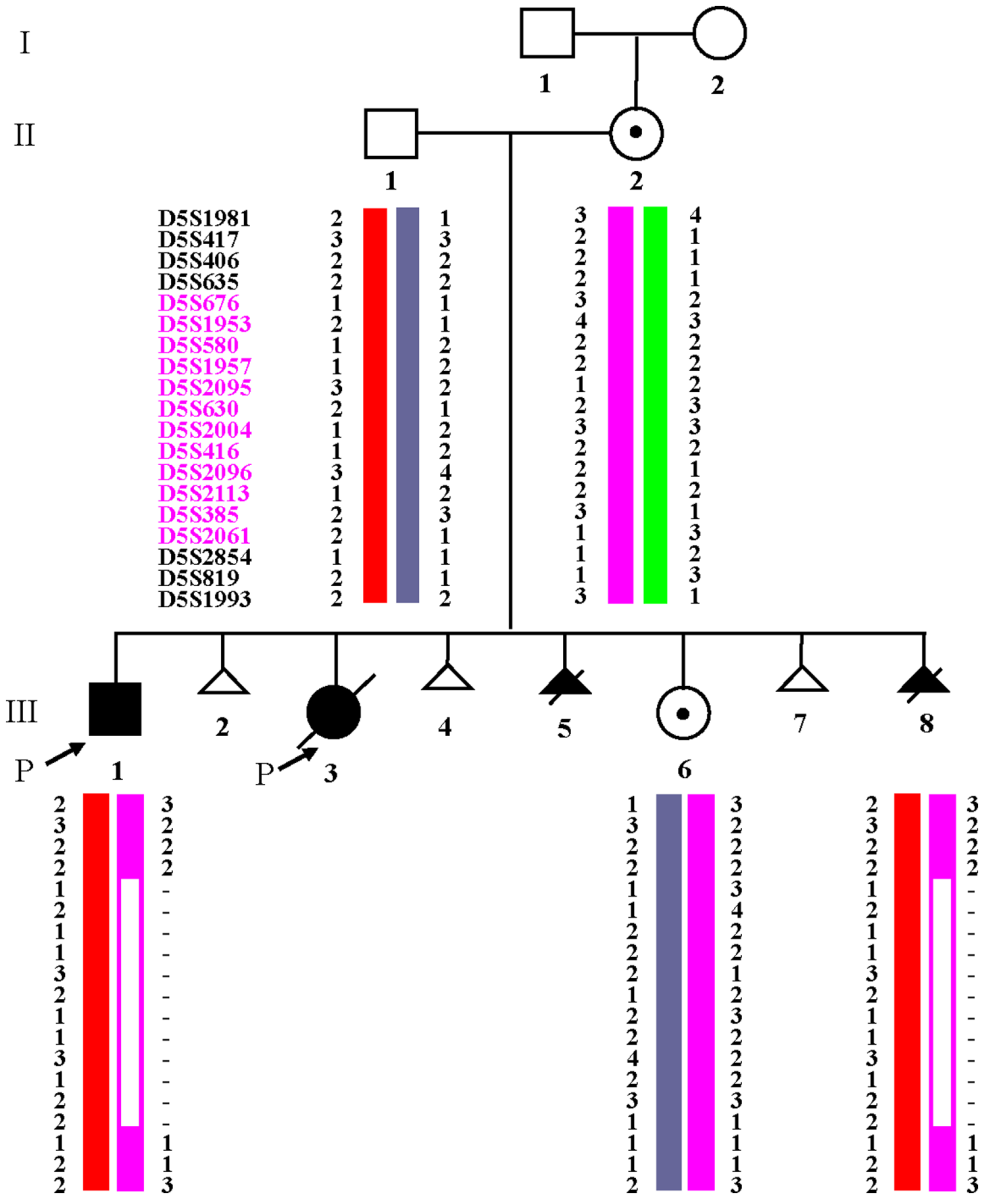
STR results. III 1 and III 8 showed an identical deletion of D5S676–D5S2061 region. III 6 inherited an identical haplotype associated with the insertion ins(11;5)from the mother.

STR testing was performed on both parents, the male proband III 1, as well as prenatally on III 6 and III 8 (Table 1 and Figure 4). In a total of 19 STR markers tested, the male proband (III 1) only obtained father’s allele apparently at D5S676, D5S1953, D5S580, D5S1957, D5S2095, D5S630, D5S2004, D5S416, D5S2096, D5S2113, D5S385 and D5S2061 (Figure 4). Because three markers (D5S406, D5S635 and D5S2854) were not informative, the encompassed interstitial deletion was estimated to be approximately 22.5 Mb between bands 5p13.3 and 5p15.33 (chr5∶7439488–29976793), which was consistent with the above chromosome finding. The same finding was observed in III 8 and confirmed 5p deletion by the prenatal chromosome analysis. STR analysis in III 6 showed a haplotype identical to the mother, which was consistent with the prenatal chromosome analysis of the apparently balanced insertion ins(11;5). This pregnancy was continued and a phenotypically normal baby girl was delivered.

### Array CGH and FISH Results

**Figure 5:**
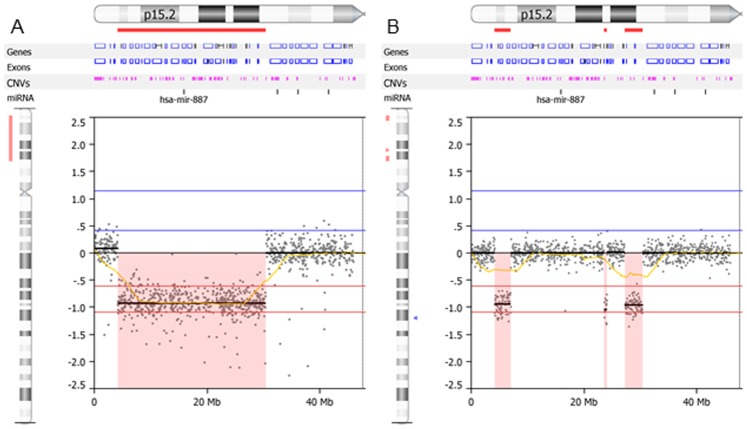
Array CGH plots to show copy number deletions on 5p observed in the male proband III 1 (A), showing a large interstitial deletion arr5p15.33p15.3(4200304–30493484)x1, and, in the normal sister III 6 (B), showing three smaller size interstitial deletions, arr5p15.33p15.31(4200304–7081712)x1,5p14.2(23642864–24156987)x1, 5p14.1p13.3(27332938–30493484)x1 with 2.89 Mb, 0.56 Mb and 3.21 Mb, respectively. All deleted segments are shaded in pink with log R ratio at ∼negative 1.0.

The male proband and his phenotypically normal sister were followed up extensively for clinical genetic evaluations. Eight years after the first genetic visit of the probands, the array CGH technology became available and it was introduced to this family. Because of the unique familial 5p- history and the rare CCRs in mother, this family agreed to have array CGH testing on both parents, the male proband III 1 (at 12 years and 9 months old) and his phenotypically normal sister III 6 (at 6 years and 1 months), for a whole genome copy number assessment. Array CGH revealed the minimal interstitial deletion in the male proband at chromosome bands 5p15.33-p13.3 to be 26.22 Mb, within the genomic interval 4200304-30493484 (Figure 5A), which was slightly larger than the deletion detected by the STR testing although was consistent with the chromosome results. The discrepancy between array CGH and STR results are most likely due to the three non-informative STR markers tested fall in breakpoints regions (Table 1). Interestingly, in the normal sister, array CGH revealed three interstitial deletions showing arr5p15.33p15.31(4200304-7081712)×1,5p14.2(23642864-24156987)×1,5p14.1p13.3(27332938-30493484)×1 with 2.89 Mb, 0.56 Mb and 3.21 Mb in size respectively (Figure 5B).The proband and the normal sister shared the distal and proximal breakpoints in 5p15.33 and 5p13.3 respectively. There were no abnormal copy number aberrations identified in both parents.

**Figure 6:**
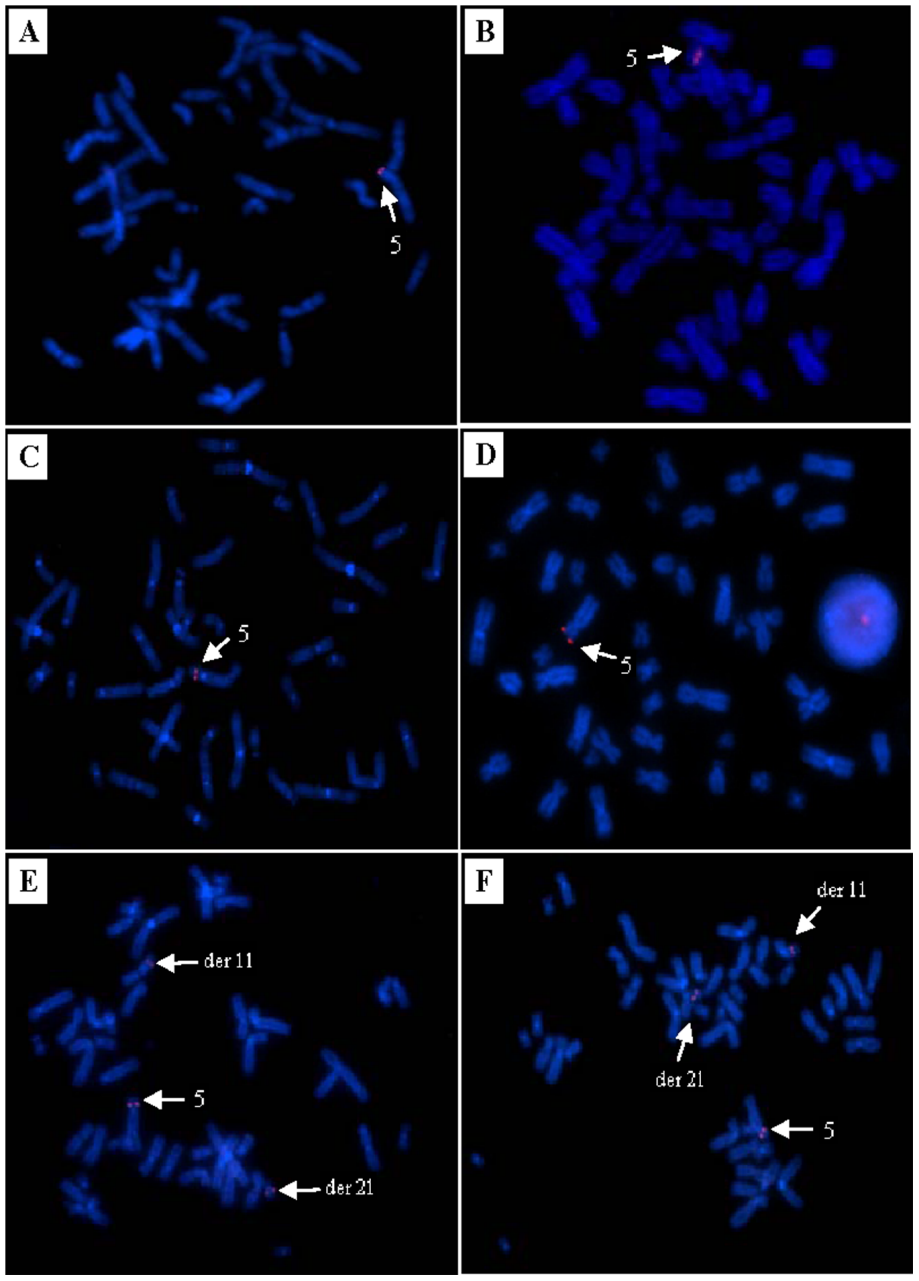
FISH examples. A, B showing the deletion examples of two BACs (RP11-59C22 and RP11-318A6) in male proband; C, D showing the deletion examples of two BACs (RP11-473F9 and RP11-428C17) in the normal sister; E, F showing one larger signal and two smaller signals of RP11-125A21 and RP11-100I1 on the normal chromosome 5, der(11) and the der(21) in the mother.

To further confirm these array CGH results, FISH analysis was carried out on the male proband III 1, the phenotypically normal sister III 6 and the mother II 2 (Table 2). In a total of 21 BAC clones on chromosome 5p tested, the male proband showed a deletion of 19 clones tested (Table 2) (from RP11-59C22 to RP11-318A6) spanning from 5p15.32-p14.1 (5126675-29130606), further confirmed the array CGH findings. In the phenotypically normal sister, FISH analysis using the same BACs showed 1) an insertion of segment from RP11-125A21 to RP11-100I1 (chr5: 6972214-27406644) (Table 2, in bold) to chromosome 11q confirming the ins(11;5) as observed in chromosome analysis, 2) a deletion of a segment from RP11-59C22 to RP11-976O8 (chr5: 5126675-6708095), and 3) a deletion of a segment from RP11-1055J13 to RP11-318A6 (chr5: 27368992-29130606) as observed in array CGH analysis. The 5p14.2 deletion detected by array CGH in the normal sister was not examined by the FISH analysis (Table 2, Figure 6) due to no available BAC clone. Interestingly, further FISH analysis using the same set of BACs on the mother, not only revealed the same ins(11;5) as described above, but also revealed two additional cryptic insertions, showing the above deleted two segments 5p15.33-p15.31 and 5p14.1-p13.3 in the daughter to be inserted to chromosome 21q specifically. In addition, one larger signal and two smaller signals of BAC RP11-125A21 and RP11-100I1 were observed on the normal chromosome 5, the der(11) and the der(21) in the mother, respectively, indicating the breakpoints of two cryptic ins(21;5) were within these two BACs correlated genomic regions (Table 2, Figure 6). Additional FISH studies using the same set of BACs involved in two ins(21;5) were conducted on the maternal grandparents, which showed no evidence of insertions or any other rearrangements. The final karyotype for the mother was revised to 46,XX,ins(11;5)(q23;p14.1p15.31),ins(21;5) (q21;p13.3p14.1),ins(21;5)(q21;p15.31p15.33),inv(7)(p22q32)dn, and the final karyotype for the daughter should be revised to 46,XX,del(5)(p13.3p14.1),del(5)(p14.2p14.2), del(p15.31p15.33),ins(11;5)(q23;p14.1p15.31)mat. FISH using the three BAC clones targeting chromosome 21q21.1–q22.11 was normal in all three individuals (III 1, II 2 and III 6) tested. Although the 5p14.2 deletion detected by array CGH in the normal sister III 6 was not further examined by the FISH analysis due to some technical limitations, this deletion could be also resulted from a maternal cryptic balanced rearrangement.

To further investigate possible clinical consequences of the three deletions in 5p in the phenotypically normal sister III 6, we carefully analyzed genes in these genomic intervals. The first interstitial deletion in III 6 expanded approximately 2.89 Mb in 5p15.31p15.33, encoding 11 known genes including *LOC340094, ADAMTS16, KIAA0947, FLJ33360, MED10, UBE2QL1, LOC255167, NSUN2, SRD5A1, PAPD7* and *MIR4278*. Of these, haploinsufficiency of *SRD5A1* along with other genes in 5p15.1 region [28] are recently reported to be associated with hypospadias and cerebellar hypoplasia in a prenatal study. Only two genes *LOC643401* and *LSP1P3*are located in the second deletion of 5p14.2 and both have not been characterized for gene function and disease association. No known genes were found in the third deletion region of 5p13.3p14.1.

## Discussion

The majority of chromosome 5p deletions are associated with CDCs [6]. Traditionally, CDCs patients are diagnosed based upon the clinical manifestations, chromosome and/or FISH analyses, or molecular based testing such as MLPA or PCR. Although CDCs is a well-defined genomic disorder, individuals with this syndrome show phenotypic and cytogenetic variability. Several genotype-phenotype studies revealed that the size of chromosome 5p deletions could vary from a single chromosome cytogenetic band to the entire chromosome 5p, and the severity or spectrum of clinical phenotypes i.e. intellectual disability and microcephaly in CDCs or 5p syndromes are related to the size and the location of the deletions [6], [29]. For example, the single chromosome band 5p15.2 deletion is reported to be responsible for dysmorphism and intellectual disability; the proximal region of 5p15.3 is associated for “cat-like” cry and speech delay [30]. These chromosomal bands are considered to be critic regions for CDCs. Genes in this region include the *SEMAF, CTNND2* [5], which involve in brain development and function, the *FLJ25076– UBC-E2* homologous gene which is highly expressed in thoracic and scalp tissues. Haploinsufficiency of these contiguous candidate genes are most likely the cause of classic spectrum in CDCs [30]. On the other hand, identical deletions of chromosome 5p14 region are reported in phenotypically normal parents and their affected children, indicating that 1) this 5p14 region is not as critic as the 5p15 region, or 2) this region encodes the possible recessive alleles resulting in reduced expressivity or variable clinical manifestations [6]. In our current study, three microdeletions resulting from the maternal CCRs were identified in the phenotypically normal sister (III 6) of the probands. Both second and third deletions are inside or overlapped with 5p14 region. Very few genes are encoded in these two deleted regions making these two genomic intervals less clinically relevant. Although there were 11 known genes (*LOC340094, ADAMTS16, KIAA0947, FLJ33360, MED10, UBE2QL1, LOC255167, NSUN2, SRD5A1, PAPD7,* and *MIR4278*) located in the first deletion in 5p15.33p15.31, only a few of them are fully characterized for gene functions and disease association. Recent whole exome sequencing study revealed that homozygous splicing mutation in *NSUN2* to be a cause of Dubowitz-like syndrome, an autosomal recessive disorder characterized by the constellation of mild microcephaly, growth and mental retardation, eczema and peculiar facies [31]; The *ADAMTS16* gene has been reported to play a role of the metalloproteinase in murine genitourinary development [32], and loss-of-function mutations of the *MED10* gene have been previously linked with WNT/GSK3β/β-Catenin pathway function [33]. Only *SRD5A1* haploinsufficiency has been speculated to be associated with cerebellar hypoplasia, hypospadias, and facial dysmorphisms in a prenatal study [28]. Our extensive clinical evaluation in the phenotypically normal sister III 6 showed no evidence of dysmorphism or intellectual disability or other apparent findings; the IQ test at age seven in this girl was 105. We postulate that these microdeletions in III 6 are either non-pathogenic benign copy number variant or only resulted in unnoticeable minor clinical manifestations. Certainly, this individual needed to be followed up and evaluated frequently in her future genetic counseling.

The majority of the CDCs patients are diagnosed in first month of life or within first year whereas the minority is diagnosed from 13 month to 47 years old [34]. Due to the limited clinical genetic testing in China, until recently, our patients were diagnosed at age 4^1/2^ and 2^1/2^ years old. Although our probands did not have the characteristic cat-like cry at birth or during their infancy (or the cat-like cry was not recognized), they did have other typical dysmorphic features as reported in CDCs and remarkable developmental delay and intellectual disability. The integrated molecule and cytogenetic testing was able to not only define the large interstitial deletion del(5)(p13.3p15.33) and confirm a clinical diagnosis in probands, but also help reveal the CCRs in the mother and therefore provide the appropriate genetic counseling to the family.

**Figure 7:**
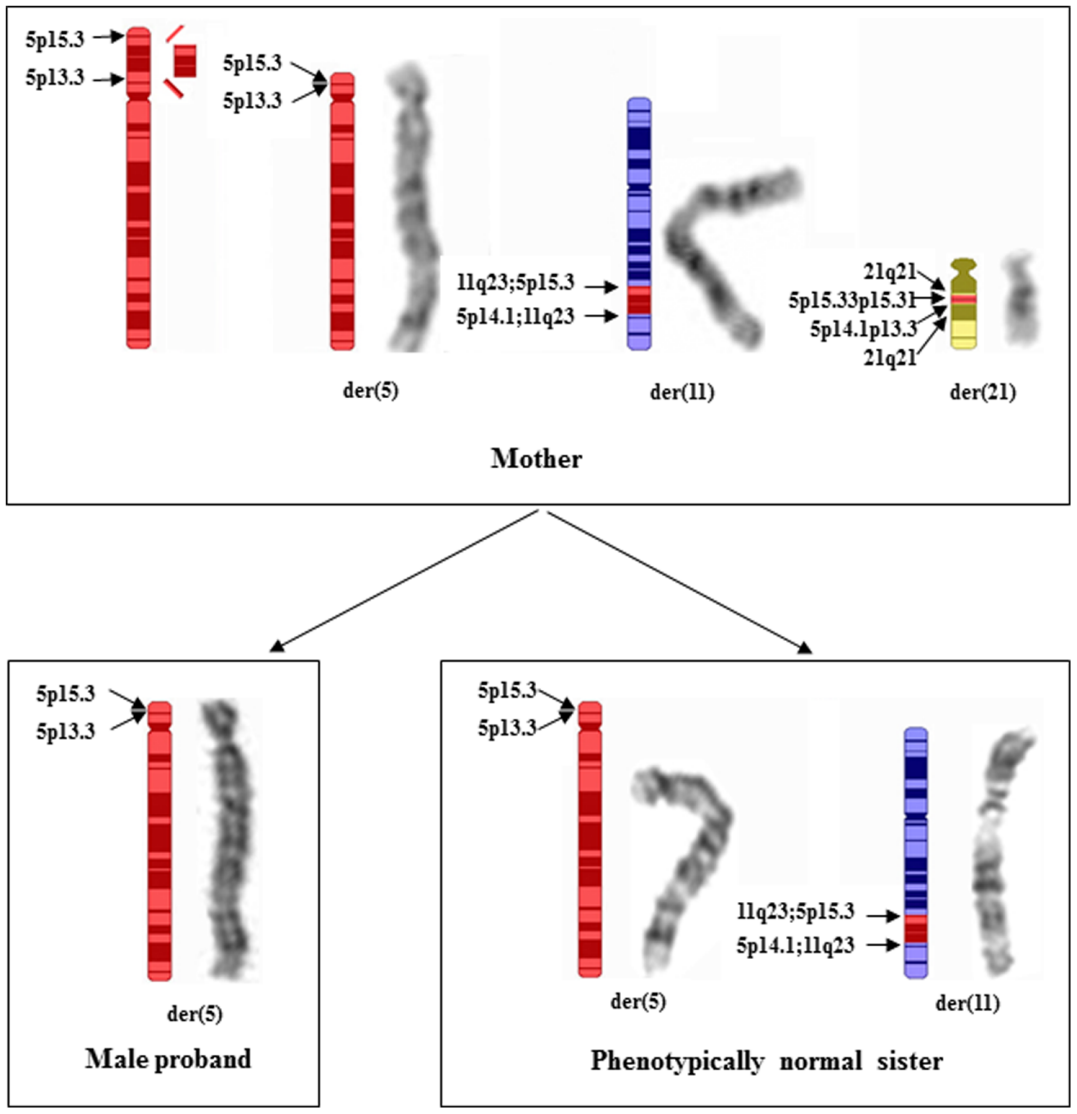
Schematic chromosome with ideogram of the maternal CCRs, illustrating the balanced rearrangements in the mother, and the terminal deletion of 5p in the male proband and as well as the apparently balanced rearrangements in the phenotypically normal sister. The average chromosome band resolution is ∼550.

CCRs are structural aberrations involving more than two chromosomes and breakpoints. Phenotypically normal individuals with CCRs often have high risk to result in multiple spontaneous abortions [13] and abnormal offspring [17]. The mother in this study had an extremely complex karyotype of 46,XX,ins(11;5)(q23;p14.1p15.31),ins(21;5) (q21;p13.3p14.1),ins(21;5)(q21;p15.31p15.33),inv(7)(p22q32)dn with apparent 11 breakpoints, involving four chromosomes which have never been reported [17]. In her total eight pregnancies, she had three first trimester miscarriages, mostly likely due to lethal chromosomal aberrations resulting from the meiotic malsegregation i.e. possible large deletions or duplications involving chromosome 7 resulted from the maternal inv(7) (Figure 7). The remaining pregnancies had abnormal chromosomal findings with majority of showing 5p deletions, which was compatible for continued pregnancies but with congenital anomalies.

Microarray based testing with significantly improved overall resolution is a more accurate clinical testing for genome-wide copy number assessment in patients with congenital anomalies [35]. Our array CGH study was able to refine the deleted segment in the proband better than STR testing, because three STR markers D5S406, D5S635 and D5S2854 were all apparently not informative. We believe that the power of microsatellite analysis is limited especially when it comes to the breakpoints involving complex rearrangements. In addition, array CGH did not detect apparent copy number aberrations in the mother, indicating that the three insertions and one pericentromeric inversion observed are all probably balanced at the array CGH testing level, and might also explain her normal phenotype. However, it did detect three microdeletions in the phenotypically normal daughter. These array findings have led to the discovery of two cryptic ins(21;5) in the mother by using additional targeted FISH analysis. Although we could not further investigate the second deletion (0.56 Mb) in III 6 by FISH due to lack of available BAC clones in 5p14.2 region, it is likely this deletion is also resulted from a maternal cryptic rearrangement. All these findings also raised questions – What had driven these germline copy number variations in the daughter? Given so many abnormal pregnancies produced, does the mother really carry all balanced rearrangements at the molecular level?

Recent studies have shown the correlations between CCRs and/or CGRs and chromothripsis in pediatric patients with congenital abnormalities [19], [20]. Although the phenotypically normal mother did not have apparent copy number aberrations on 5p, this could be due to limited resolution (180 K total oligo coverage) of the oligo array CGH applied in this study. We believe that, the extremely complex CCRs in the mother might have resulted from chromosome 5p chromothripsis, and that was exactly what had driven so many affected individuals and pregnancies in this family. The microdeletions observed in the daughter are the direct evidence of such underline mechanism, although high resolution sequencing base testing is needed to confirm such speculation.

Without prenatal integrated molecular and cytogenetic testing, consequently, this woman would have produced four children and all with 5p deletion syndrome or CDCs (Figure 2). The clinical significances of the three microdeletions identified in the phenotypically normal sister were not clear, although they were most likely representing non-pathogenic based on current available genomic variation databases; however, these findings will be valuable for her future genetic counseling.

In summary, we are reporting a very unique familial chromosome CDCs/5p deletion syndrome, resulted from unusual maternal *de novo* CCRs and/or chromosome 5p chromothripsis. The integrated molecular and cytogenetic testing not only fully characterized the maternal CCRs, but also enabled unveiling this familial event and providing accurate post- and pre-natal diagnosis as well as appropriate genetic counseling. Certainly, further sequencing based testing is needed to help determine whether chromothripsis also exists in phenotypically normal individuals in this family.
